# EXERCISE ACUTELY IMPROVES COGNITION IN HEALTHY OLDER ADULTS: THE ROLE OF AROUSAL

**DOI:** 10.1093/geroni/igz038.3079

**Published:** 2019-11-08

**Authors:** Pearl N Cummins, James Kent, Timothy Weng, Vincent Magnottta, Gary Pierce, Michelle Voss

**Affiliations:** 1 San Diego State University, San Diego, California, United States; 2 University of Iowa, Iowa City, Iowa, United States; 3 University of Texas, Austin, Texas, United States; 4 University of Iowa, Iowa City, United States

## Abstract

Previous researchers have reported that aerobic exercise improves cognition in older adults; however, few researchers have examined the role of arousal on improvements in cognition after exercise. The purpose of this study was to understand how changes in arousal acutely affect changes in cognitive performance after a single session of light compared to moderate intensity aerobic exercise. Cognitively normal older adults (N = 34) were enrolled in a randomized controlled trial where they were asked to complete the N-back task with faces, a cognitive task used to test working memory, in an fMRI scanner. On separate days, the task was completed before and 15 to 20 minutes after light and moderate intensity exercise. An intervention was also completed, but our question focuses on the acute effects of exercise rather than training. Arousal was measured before and after exercise through a questionnaire and a direct measure of physiological activation of the sympathetic nervous system with galvanic skin response (GSR). On average, resting GSRs decreased from pre- to post-exercise scan; however, the change was not statistically significant. The decrease in arousal after light exercise indicated that older adults had decreased sympathetic activity after both light and moderate intensity exercise. By contrast, N-back task performance improved most after moderate compared to light intensity exercise. Together, evidence that sympathetic activity tended to decrease generally for both intensities, whereas cognitive improvements were more specific, suggests that changes in arousal at rest were not a critical factor connecting exercise and improved working memory in this study.

